# Expression in *A. thaliana* and cellular localization reveal involvement of *BjNRAMP1* in cadmium uptake

**DOI:** 10.3389/fpls.2023.1261518

**Published:** 2023-10-12

**Authors:** Ting Li, Yicun Li, Jiaqi Wang, Jiashi Peng, Lili Liu, Lichao Deng, Dawei Zhang, Mingli Yan

**Affiliations:** ^1^ Hunan Key Laboratory of Economic Crops Genetic Improvement and Integrated Utilization, School of Life and Health Science, Hunan University of Science and Technology, Xiangtan, China; ^2^ Hunan Research Center of Heterosis Utilization in Rapeseed, Crop Research Institute, Hunan Academy of Agricultural Sciences, Changsha, China

**Keywords:** *Brassica juncea*, cadmium, cellular localization, manganese, uptake

## Abstract

Although *Brassica juncea* has demonstrated potential as a hyperaccumulator crop, it was not entirely clear how cadmium (Cd) accumulates in plants. Here, we found that BjNRAMP1 (Natural Resistance-Associated Macrophage Protein 1) plays a crucial role in the accumulation of Cd and manganese (Mn) through its expression in yeast and *Arabidopsis thaliana*. The high concentration of Cd exposure could induce the expression of *BjNRAMP1*. The ectopic expression of *BjNRAMP1* in yeast led to higher accumulation of Cd and Mn compared to the vector control. BjNARAMP1 was localized to the plasma membrane and expressed in the vascular system of roots, leaves, and flowers. The overexpression of *BjNRAMP1* in *A. thaliana* resulted in an increased accumulation of Cd in both roots and shoots, which inhibited the normal growth of transgenic lines. Moreover, Mn uptake in roots was activated by the increase in Cd stress. Together, our results indicated that BjNRAMP1 significantly contributes to the uptake of Mn and Cd in *B. juncea.*

## 1 Introduction

Agricultural soils are under threat of cadmium (Cd) contamination from natural processed and anthropogenic activities, leading to human health risks after chronic dietary exposure (Zhao et al., 2015; Zhao et al., 2022). Among several strategies to prevent the negative effects of Cd, crop breeding or engineering is the key to either increasing the Cd accumulation in nonedible parts for phytoremediation or reducing that in edible parts for safer food (Clemens et al., 2013; Clemens, 2019). Thus, understanding the mechanisms of Cd uptake, translocation, and detoxification provides potential targets for marker-assisted breeding or genetic engineering of crops that accumulated either high or low levels of Cd (Zhao and Wang, 2020).

As a toxic element, Cd was inadvertently taken up into root cells and transported to other plant tissues via membrane transporters for essential or beneficial nutrients. NRAMP5 (Natural Resistance Associated Macrophage Proteins 5) has been identified as a major Cd transporter in rice. The use of RNAi or CRISPR/Cas9 to knock out *OsNramp5* led to a significant reduction in the Cd concentration in the root, shoot, and grain (Sasaki et al., 2012; Tang et al., 2017). OsNRAMP1 was located to the plasma membrane of endodermis and pericycle cells, and it is involved in the Cd uptake and transport within the cells. The higher expression of *OsNRAMP1* in the roots resulted in an increase in Cd accumulation in the shoots. The growth of yeast expressing *OsNRAMP1* was impaired in the presence of Cd compared with yeast transformed with an empty vector (Takahashi et al., 2011). However, the expression of *OsNRAMP1* in *Arabidopsis thaliana* conferred tolerance phenotype with increased Cd accumulation in the root and shoot (Tiwari et al., 2014). *OsNRAMP1* expression could be induced by both Cd treatment and Fe deficiency. The knockout of *OsNRAMP1* resulted in significant decreases in root uptake of Cd and Mn, while the effect was less than in those from knockout of *OsNRAMP5* (Chang et al., 2020).

*Brassica juncea* is an important vegetable- and oil-use crop worldwide and exhibits the potential to tolerate and accumulate Cd, and it can be considered as a Cd hyperaccumulator plant (Kang et al., 2021; Zeremski et al., 2021). Our previous study suggested that a higher concentration of Cd could induce the expression of *BjNRAMP1*, while its role in Cd transport in *B. juncea* is hitherto unknown (Zhang et al., 2021). Herein, we found that BjNRAMP1 was localized to the plasma membrane and played a vital role in Cd accumulation through its expression in yeast and *A. thaliana.*

## 2 Materials and methods

### 2.1 Plant materials and growth condition

The *B. juncea* cultivar “Purple Leaf Mustard” was grown in Cd-contaminated soils as described previously (Zhang et al., 2021).

The *A. thaliana* plants were grown in soil to generate the T3 homozygous transgenic lines. The wild type (Col-0) and T3 lines were cultured in 1/2MS medium containing 0, 10, 30, and 50 μM CdCl_2_ for 10 days at 22°C under a 16-h light/8-h dark cycle.

### 2.2 Cloning and sequence analysis of *BjNRAMP1*

Total RNA was extracted and cDNA synthesis was performed using a Thermo Fisher Scientific RevertAid First Strand cDNA Synthesis Kit. Full-length cDNA encoding *BjNRAMP1* was amplified through PCR using gene-specific primers (**Table S1**). The amplified products of the PCR were cloned into the pMD19-T vector (Takara) and sequenced via the Sanger method. The amino acid sequences of BjNRAMP1 and the other related species were employed to perform multi-sequence alignment using the ClustalW with default parameters in MEGA7. The phylogenetic tree was subsequently constructed using the neighbor-joining approach (no. of bootstrap replications = 1,000) implemented in MEGA7.

### 2.3 Yeast expression assays

Confirmed *BjNRAMP1* CDS in the pMD19-T vector were digested with restriction enzymes and inserted into the pYES2 expression vector. After sequencing verification, the empty and target vector was transferred into the Cd-sensitive yeast mutant Δ*yap1* and wild-type Y252, according to the yeast transformation and phenotype identification methods provided by Peng et al. (2017). In Δ*yap1*, the vacuolar Cd sequestration was decreased already, resulting in hypersensitivity to Cd relative to wild-type cells (Wemmie et al., 1994). Meanwhile, the plasmids were also extracted and transformed into the zinc-sensitive yeast mutant Δ*zrc1* and its wild-type CM100, and copper-sensitive yeast mutant Δ*cup2* and its wild-type BY4741 using the lithium acetate method as we described before (Zhang et al., 2022).

### 2.4 Generation of transgenic plants

The *BjNRAMP1* gene fragment was ligated into pEGOEP35S-H, and transformed into *A. thaliana* wild-type Col-0 by the *Agrobacterium tumefaciens* GV3101 dip flowering method. The transformed lines were screened for hygromycin resistance and verified by sequencing. T3 generations of homozygous transgenic lines were used for phenotype identification.

A 1,917-bp promoter sequence located upstream of the *BjNRAMP1* start codon was obtained by PCR amplification. The pBWA(V)BII-GUS vector was cleaved with enzymes *BsaI* and *Eco31I* and the target fragment was ligated to the linear vector using homologous recombination to construct the *proBjNRAMP1*:*GUS* fusion protein expression vector. The *proBjNRAMP1:GUS* vector was transformed into the *A. thaliana* wild type (Col-0).

### 2.5 Subcellular localization of BjNRAMP1

To determine the localization of the *BjNRAMP*1 gene in plant cells, the full-length coding sequence without a stop codon of the *BjNRAMP*1 gene was amplified by RT-RCR and transformed into both *A. thaliana* protoplasts and tobacco (*Nicotiana benthamiana*) leaf epidermal cells using a plant expression binary vector pEGOEP35S-H with a GFP fluorescent tag (pEGOEP-H- *35S:BjNRAMP1*-*GFP*). The preparation of protoplasts and their transformation into *A. thaliana* was performed following the polyethylene glycol method (Peng et al., 2017). Meanwhile, the *Agrobacterium tumefaciens* GV3101 solutions with the pEGOEP-H- *35S*:*BjNRAMP1*-*GFP* vector were injected into the 3-week-old tobacco leaf epidermal cells. After incubation of *A. thaliana* protoplasts at 23℃ in the dark for 20 h and growth of tobacco for 2 days in dark, the GFP fluorescence was examined using a confocal microscope (FV1000; OLYMPUS). The localization marker of the cell plasma membrane is AtPIP2A-RFP.

### 2.6 qRT-PCR analysis of *BjNRAMP1*

Tissues from *B. juncea* and *A. thaliana* were collected and immediately frozen in liquid nitrogen with three biological replicates. First-strand cDNA was synthesized using a Tsingke Goldenstar RT6 cDNA Synthesis Kit ver.2 (Tsingke, Beijing, China). qRT-PCR was performed using SYBR Premix Ex TaqII on a Bio-Rad CFX 96 Real-Time Detection System as described previously (Zhang et al., 2020a).

### 2.7 Metal measurement in *A. thaliana* and yeast

For metal measurement in *A. thaliana* lines, the harvested plants were washed with distilled water, followed by drying at 75℃ until completely dehydrated. Plants were ground to fine powder, digested in 70% nitric acid at 100℃ for 2 h, and diluted with ultrapure water for metal determination.

For metal measurement in yeast, yeast cells that were transformed with empty vectors and *BjNRAMP1* were cultured to OD = 0.8 with 20 μM CdCl_2_ solution, treated, and incubated for 24 h. The cells were collected by centrifugation and washed with sterile water and 2 mmol/L EDTA. After washing well, the cells were dried at 70°C and subsequently nitrated with 70% concentrated nitric acid and diluted. The concentrations of Cd and other elementals in all samples were determined by inductively coupled plasma-mass spectrometry (ICP-MS).

## 3 Results

### 3.1 Higher concentration of Cd stress induced the expression of *BjNRAMP1*

**Figure 1:**
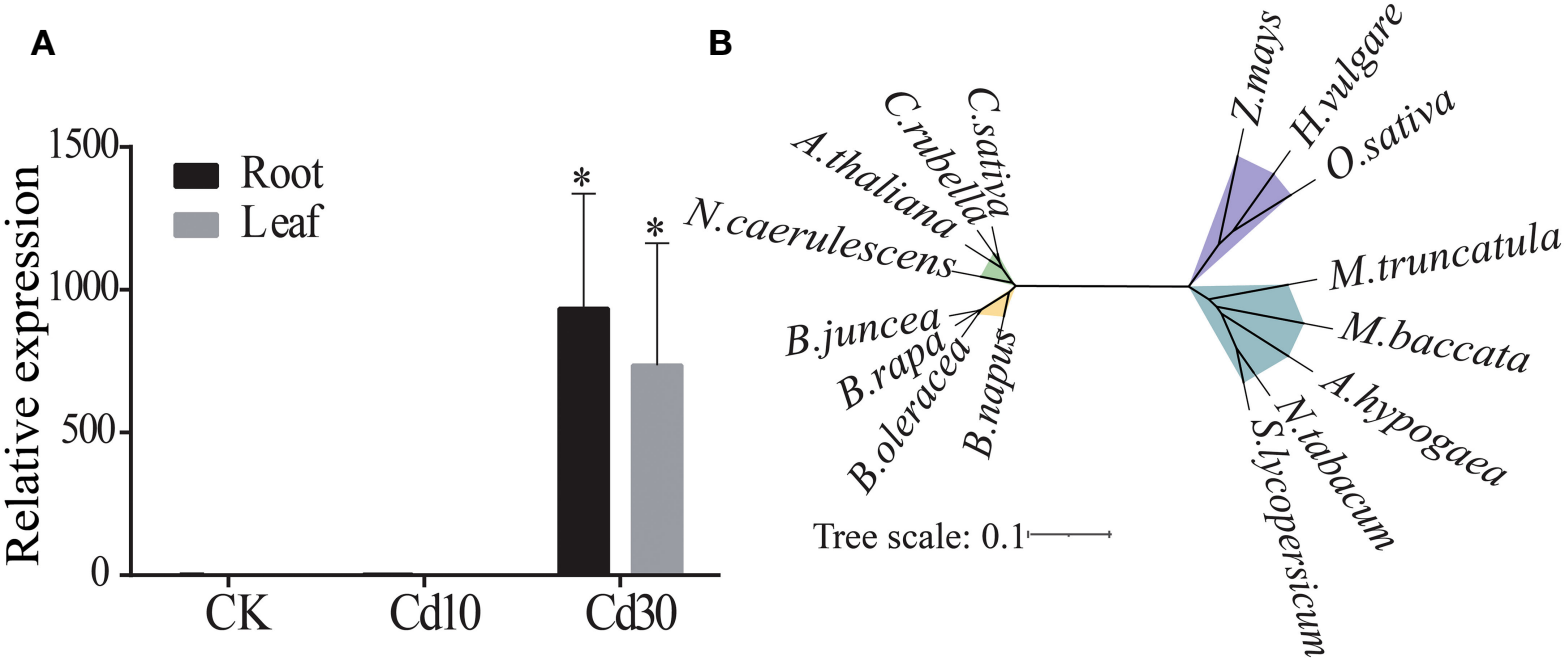
Expression and phylogenetic analysis of *BjNRAMP1*. **(A)** Expression of *BjNRAMP1* in root and leaf under different Cd treatments (CK, 0 mg/kg; Cd10, 10 mg/kg; Cd30, 30 mg/kg). The relative expression value under Cd30 was compared with that CK and Cd10 using *t*-test, * represents *p* < 0.05. **(B)** Phylogenetic analysis of the NRAMP1 protein. The sequences could be found in the NCBI database with the accession numbers: *A thaliana* (NP_178198.1), *A hypogaea* (XP_025612907.1), *B napus* (XP_013726991.2), *B oleracea* (XP_013588655.1), *B rapa* (XP_009106714.1), *C sativa* (XP_010472867.1), *C rubella* (XP_006301485.1), *H vulgare* (XP_044960708.1), *M. baccata* (AAU00158.1), *M. truncatula* (KEH35319.1), *N. caerulescens* (JAU89736.1), *N. tabacum* (XP_016485353.1), *O. sativa* (NP_001389971.1), *S. lycopersicum* (NP_001234318.1), and *Z. mays* (XP_008670084.1).

Previously, we reported that the *BjNRAMP1* was upregulated when *B. juncea* was exposed to high concentrations of Cd using RNA-Seq (Zhang et al., 2021). Herein, qRT-PCR result confirmed that the expression of *BjNRAMP1* was silenced under either control or 10mg/kg Cd stress, but induced in both leaves and roots when plants were under 30 mg/kg Cd stress, suggesting that *BjNRAMP1* was sensitive to higher concentration of Cd exposure (**Figure 1A**).

Cloning and sequence analysis indicated that *BjNRAMP1* was 1,566 bp in length, encoding a protein with 521 amino acid residues with 12 predicted transmembrane domains (**Figure S1**). Multiple alignments indicated that the BjNRAMP1 exhibited high identities to that in *B. rapa* (99%), *B. oleracea* (98.84%), *B. napus* (99%), and *A. thaliana* (85.90%). The phylogenetic analysis showed that *BjNRAMP1* was closely related to its homologous genes in *Brassica*, including *B. rapa*, *B. oleracea*, and *B. napus*, but evolutionarily far away from that in Poaceae, such as *O. sativa*, *Z. mays*, and *H. vulgare* (**Figure 1B**).

### 3.2 Ectopic expression of *BjNRAMP1* in yeast promotes Cd and Mn uptake

**Figure 2:**
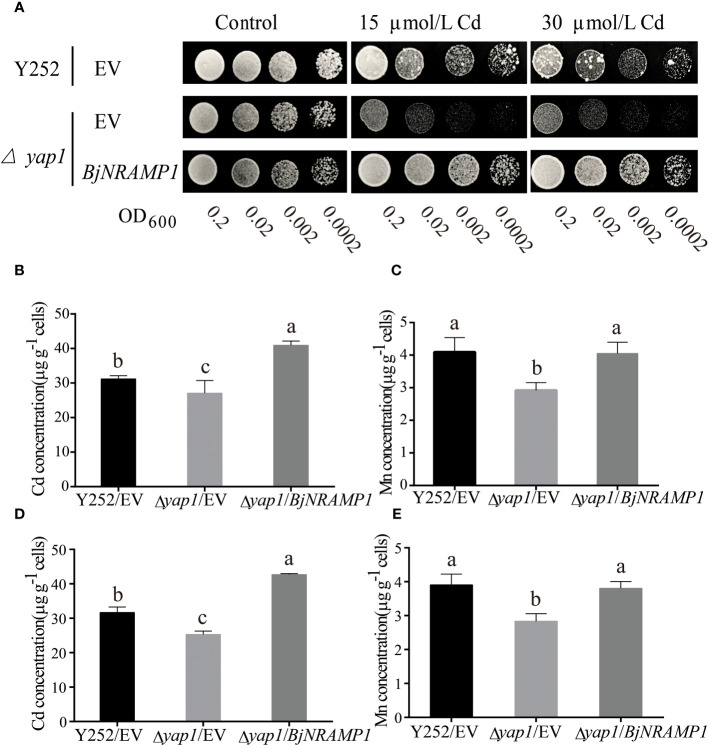
Functional characterization of BjNRAMP1 in yeast. **(A)** The Cd transport activity assay of BjNRAMP1 in yeast. Growth of yeast mutant strain *Δyap1* expressing *BjNRAMP1* or empty vector (pYES2) and yeast wild strain Y252 expressing empty vector (pYES2) in a medium containing different concentrations of Cd. Cd **(B)** and Mn **(C)** concentration in yeast with 20 μM CdCl_2_ treatment for 6 h. Cd **(D)** and Mn **(E)** concentration in yeast with 20 μM CdCl_2_ treatment for 12 h. Data were analyzed by one-way ANOVA using GraphPad Prism 9.3.1. Different letters indicate significant difference at *p* < 0.05 according to Tukey’s multiple comparisons tests.

To investigate the role of *BjNRAMP1* in metal accumulation, the gene was expressed in the yeast heterologous system. Ectopic expression in Cd-sensitive mutant Δ*yap1* showed that the growth of transformed yeasts with *BjNRAMP1* was enhanced as compared with the control with empty vector in the presence of 15 and 30 μM Cd (**Figure 2A**). The concentration of Cd and Mn of yeasts transformed with *BjNRAMP1* was significantly higher than that in control when yeasts were under 20 μM Cd treatment for 6 and 12 h (**Figures 2B–E**). After 6 h of Cd treatment, the Cd concentration in yeasts transformed with *BjNRAMP1* (40.93 μg/g) was not only 1.5-fold higher than that in Δ*yap1* with empty vector (27.08 μg/g), but also significantly higher than that in wild type (31.18 μg/g). As to Mn, the *BjNRAMP1* transformed yeasts (4.05 μg/g) exhibited a similar level of Mn concentration with wild type (4.09 μg/g), but significantly higher than that in Δ*yap1* with the empty vector (3.12 μg/g). Additionally, the results were also observed when yeasts were treated with 20 μM Cd for 12 h, suggesting that BjNRAMP1 could facilitate the uptake of Cd and Mn.

However, no significant increase in the concentration of Zn, Cu, and Fe was measured in *BjNRAMP1* transformed yeasts as compared with the empty vector (**Table S2**). Meanwhile, no significant phenotypic differences were observed when *BjNRAMP1* is expressed in Zn- and Cu-sensitive mutants (**Figure S2**), indicating that BjNRAMP1 might be one of the major transporters of Cd and Mn, but not that of Zn and Cu.

### 3.3 BjNRAMP1 is localized to the plasma membrane in the vascular system

**Figure 3:**
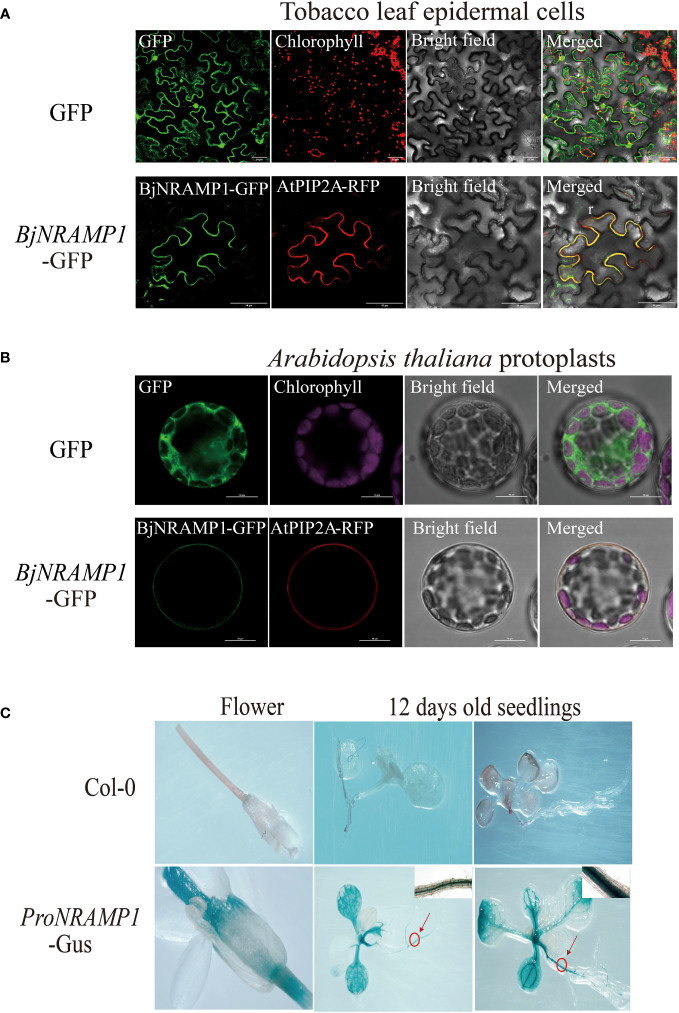
Subcellular localization and histochemical staining of BjNRAMP1. **(A)** Subcellular localization of BjNRAMP1 in tobacco epidermal leaf cells. The protein with GFP shows green signals. The plasma membrane marker AtPIP2A-RFP shows red signals. Scale bar, 50 μm. **(B)** Subcellular localization of BjNRAMP1 in *A thaliana* protoplasts. Scale bar, 10 μm. **(C)** Histochemical staining of GUS activity of *proBjNRAMP1*-*GUS* transgenic plants at the seedling and flowering stage. The image in the upper right is an enlarged view of the root section at the part with a red circle.

To determine the subcellular localization of BjNRAMP1, the gene was fused with GFP fluorescent tag and under the regulation of the CaMV 35S promoter (*35S*:*BjNRAMP1*-*GFP* vector), and then the transiently expressed vector was transformed into both tobacco leaf epidermal cells and *A. thaliana* protoplasts (**Figures 3A, B**). In the tobacco leaf epidermal cells, the green fluorescence of fusion proteins (BjNRAMP1-GFP) was observed at the plasma membrane and co-localized with the plasma membrane marker AtPIP2A-RFP (**Figure 3A**). Despite the fact that the fluorescence intensity of BjNRAMP1-GFP in *A. thaliana* was slightly weaker than that in tobacco, the co-localized BjNRAMP1-GFP and plasma membrane marker AtPIP2A-RFP was still found in its protoplasts, confirming that BjNRAMP1 is localized to the plasma membrane (**Figure 3B**). Histochemical staining of GUS activity driven by the promoter of *BjNRAMP1* (*proBjNRAMP1:GUS*) showed that *BjNRAMP1* was mainly expressed in the vascular system of roots and leaves at the seedling stage, as well as stigma at the flowering stage (**Figure 3C**).

### 3.4 Overexpression of *BjNRAMP1* increase the Cd accumulation

**Figure 4:**
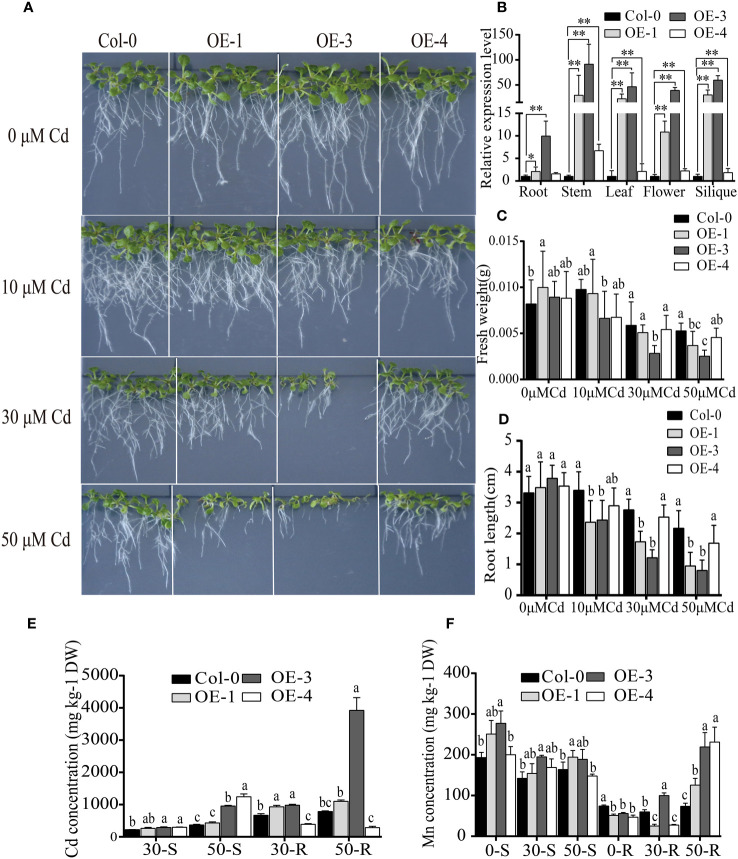
Functional analysis of BjNRAMP1 in *A thaliana*. **(A)** Phenotypes of overexpression lines at different concentrations of Cd treatment. **(B)** Relative expression levels of *BjNRAMP1* in overexpressing lines and wild type at different tissues. Col-0 represents Colombian wild-type *A thaliana*, and OE-1, OE-3, and OE-4 represent *BjNRAMP1* overexpression lines. *t*-test was performed between the wild type and each overexpression line. Measurement of root length **(C)** and fresh weight **(D)** in overexpression lines under different Cd concentrations. Determination of Cd **(E)** and Mn **(F)** content in the shoot and root of overexpression lines treated with different Cd concentrations. 0, 30, and 50-S or -R indicate shoots and roots of *A thaliana* under 0, 30, and 50 μM Cd treatment, respectively. DW indicates dry weight. Data were analyzed by one-way ANOVA, followed by comparisons of means of wild type and overexpression lines using Tukey’s multiple comparisons test. Different letters indicate significant difference at *p* < 0.05.

To further verify the function of *BjNRAMP1*, its coding sequence was driven by the CaMV 35S promoter and transformed into *A. thaliana* Col-0 generating the overexpression lines (**Figure 4**). After qRT-PCR confirmation, three lines, OE-1 (2.09- to 29.86-fold), OE-3 (9.96- to 59.30-fold), and OE-4 (1.59- to 6.73-fold), which exhibited significantly higher expression than the Col-0, were selected for further analysis (**Figure 4B**). In the presence of different concentrations (10, 30, and 50 μM) of Cd, the growth of *BjNRAMP1* overexpressing lines was significantly inhibited, displaying smaller seedling size (**Figure 4A**), lower fresh weight (**Figure 4C**), and shorter root length (**Figure 2D**). For example, no significant differences in phenotype (*p* > 0.05) were observed between the non-transformed line Col-0 and the overexpressing line OE-3 when Cd was absent (0 μM). However, the fresh weight and root length of OE-3 were dramatically decreased approximately 29.7% and 32.7% (*p* < 0.05), respectively, as compared with Col-0 under 10 μM Cd treatment. The inhibition effect was increased with the increase in the concentration of Cd, resulting in 50.8% and 54.5% decrease in fresh weight, as well as 52.1% and 71.7% decrease in root length observed under 30 and 50 μM Cd treatment, respectively.

The Cd concentration in the shoots and roots of the overexpression line OE-3 was significantly higher than that of the non-transformed line Col-0 (**Figure 4E**). Although significantly more Cd was accumulated in the shoots of OE-4, the Cd concentration in the roots was decreased. Together, these results suggested that BjNRAMP1 could increase the Cd accumulation in plants. Meanwhile, the Mn concentration in the roots of overexpression lines was significantly lower under normal growth conditions (Cd0), but significantly higher than that in Col-0 when plants were under 50 μM Cd stress, indicating that high Cd stress could promote the Mn uptake in the roots of overexpression lines (**Figure 2G**). In accordance with the findings in yeast, no significant increase in concentration of Zn, Cu, and Fe was observed in the *A. thaliana* overexpression lines as compared to the wild type (**Table S3**).

## 4 Discussion

Although the role of OsNRAMP1 in rice has been well characterized (Tiwari et al., 2014; Chang et al., 2020), the BjNARAMP1 has not been established unequivocally due to the lack of functional analysis either in yeast or in plant. In the present study, our focus was to elucidate the role of BjNRAMP1 in Cd transport and accumulation using yeast and *A. thaliana* systems. We found that the exposure to higher concentration of Cd could induce the expression of BjNRAMP1, leading to significantly higher transcript levels in both leaves and roots (**Figure 1A**). Similar results were also observed in rice (Chang et al., 2020) and *M. hupehensis* (Zhang et al., 2020b), suggesting that a similar positive feedback mechanism might have evolved since it would lead to increased Cd uptake. The heterologous expression of *BjNRAMP1* in yeast showed that BjNRAMP1 could promote the uptake of both Cd and Mn (**Figure 2**), which agrees with the results in rice (Takahashi et al., 2011; Tiwari et al., 2014; Chang et al., 2020). However, when *BjNRAMP1* is overexpressed in Cd-, Zn-, and Cu-sensitive yeast, no significant increase in the concentration of Zn and Cu (**Table S2**) was observed, and there were no significant differences in phenotype (**Figure S2**). These results suggest that BjNRAMP1 was capable of uptake of both Cd and Mn, rather than Zn and Cu. A previous study found that the knockout of NRAMP1 had no significant effects on the concentrations of Fe, Zn, or Cu in rice (Chang et al., 2020), which was consistent with our results in yeast and *A. thaliana* (**Table S3**).

Previous studies have shown that NRAMP1 was a plasma membrane-localized protein that contributed to Cd and Mn uptake in rice (Tiwari et al., 2014; Chang et al., 2020). In *A. thaliana*, it was observed that NRAMP1 has undergone dynamic cycling between the plasma membrane and endosomal compartments, and overexpression of *AtNRAMP1* conferred Cd sensitivity (Castaings et al., 2021; Li et al., 2022). In our study, the results of cellular analysis in both tobacco and *A. thaliana* revealed that BjNRAMP1 was localized to the plasma membrane (**Figures 3A, B**). GUS staining results indicated that *BjNRAMP1* was mainly expressed in the vascular system of roots and leaves, and stigma of *A. thaliana* (**Figure 3C**).

Overexpression of *BjNRAMP1* in *A. thaliana* significantly enhanced the accumulation of Cd in roots and shoots, and inhibited the growth of transgenic lines as compared with wild type in the presence of Cd stress (**Figure 4**). In general, the inhibition effect was increased with the increase in Cd treatment concentration and the expression level of *BjNRAMP1*. It was observed that significantly more Cd accumulated in the shoots but less Cd accumulated in the roots of OE-4 under Cd stress (**Figure 4E**). *BjNRAMP1* was expressed relatively higher in the stem (6.73-fold) of OE-4 than in the roots (1.5-fold). We assumed that BjNRAMP1 might also be involved in the translocation of Cd from roots to shoots in OE-4, thus reducing the accumulation in roots, conferring better Cd resistance ability than OE-1 and OE-3 (**Figures 4C, D**). In the study of Tiwari et al. (2014), the expression of *OsNRAMP1* in *A. thaliana* enhanced the Cd accumulation and tolerance, demonstrating the role of OsNRAMP1 in xylem-mediated loading. Therefore, further detailed analyses, such as BjNRAMP1-GFP immunostaining in cross-sections across different tissues and microfocus X-ray fluorescence, are still required to determine its tissue localization and clarify its role in Cd uptake and translocation.

Significantly more Mn accumulated in the roots of overexpression lines than that in wild type when plants were under the higher concentration of Cd stress, suggesting that Mn uptake in roots was activated in addition to the increase in Cd accumulation (**Figure 4F**). Furthermore, overexpression of *BjNRAMP1* did not change the ratio of Mn translocated from roots to the shoots, indicating that BjNRAMP1 was involved primarily in the uptake of the Mn into the roots, rather than in the root-to-shoot translocation.

## 5 Conclusion

Together, our results demonstrate that BjNARAMP1 was localized to the plasma membrane, mainly expressed in the vascular system of roots and leaves, and stigma, and involved primarily in the uptake of the Cd and Mn into the roots.

## Data availability statement

The raw data supporting the conclusions of this article will be made available by the authors, without undue reservation.

## Author contributions

TL: Writing – original draft, Investigation, Visualization. YL: Writing – original draft, Investigation. JW: Writing – original draft, Investigation. JP: Writing – original draft, Investigation, Resources. LL: Writing – original draft, Investigation, Resources. LD: Investigation, Writing – original draft. DZ: Writing – review & editing, Funding acquisition, Project administration, Writing – original draft. MY: Writing – review & editing, Project administration, Supervision.
