# Supplementary material for: Expression in A. thaliana and cellular localization reveal involvement of BjNRAMP1 in cadmium uptake
Source: Front Plant Sci. 2023 Oct 12;14:1261518. doi: 10.3389/fpls.2023.1261518 (PMC10600467; doi:10.3389/fpls.2023.1261518)
Supplement: Supplementary file 1 [file DataSheet_1.docx]

**Table S1. Primers used in the present study**

| Primer name | Primer sequences(5＇-3＇) | Enzyme | Purpose of primer |
| --- | --- | --- | --- |
| BjNRAMP1-F | ATGGCTATGGCGGATGCTTCTG |  | BjNRAMP1-CDS cloning |
| BjNRAMP1-R | ATCAGCATTGGATGTAGACGCT |  |  |
| ProBjNRAMP1-F | GGATCCATTTCCCCTGCCAATTATGTAGT |  | BjNRAMP1 gene promoter sequence cloning |
| ProBjNRAMP1-R | CCATGGCCTGAGGCGAGCTTTAAGGA |  |  |
| ProBjNRAMP1-GUS-F | CGCCTGCAGGTCTAGATAGAAATTATGCCACAATTTTAGAATTACATTTATATTTTAGTTT | *Bsa*I | ProBjNRAMP1*-*GUS vector construct |
| ProBjNRAMP1-GUS-R | CCCTCAGATCTACCATCGCACCTGAGGCGAGCTTTAAGGAGATTTTATG | *Eco*31I |  |

| 35seq-F | AGGAAGTTCATTTCATTTGGAGAGAACACGGGGGAC |  | GUS staining vector colony PCR primer |
| --- | --- | --- | --- |
| GUS-R | CCATGGCCTGAGGCGAGCTTTAAG |  |  |
| BjNRAMP1-G-R | CTGTTGATTCTACTTGAAGATGTCCAA |  | GUS staining recombinant plasmid sequencing primer |
| *35S::*BjNRAMP1-eGFP-F | ACTAGGGTCTCGCACC ATGGCTATGGCGGATGCTTCTGG | *Bsa*I | *35S::*BjNRAMP1-eGFP vector construct |
| *35S::*BjNRAMP1-eGFP-R | ACTAGGGTCTCTCGCC GAGATTATCAGCATTGGATGTAGAC | *Bsa*I |  |
| 35S-F | CACGGGGGACTCTTGCCACC |  | Subcellular location-based expression vector colony PCR primers |
| eGFP-cx | GACACGCTGAACTTGTGG |  |  |
| BjNRAMP1-C-R | GGGGGTTAAATCCGAGGCAT |  | Sequencing primers for subcellular localization of recombinant plasmids |
| BjNRAMP1-C2-R | CCAAGATTGGCAGCCAGAGA |  |  |
| qPCR- BjNRAMP1-F | GGATTGGTGTTCTTCTTACAGG |  | qRT-PCR |
| qPCR-BjNRAMP1-R | GCGTCTGGCTTTGAGTAGTG |  |  |
| Actin-F | GGTAACATTGTGCTCAGTGGTGG |  | Internal reference primer |
| Actin-R | AACGACCTTAATCTTCATGCTGC |  |  |

**Table S2 Effect of Cd treatment on metal accumulation in yeasts**

| Yeasts | 6 hours | | |  | 12 hours | | |
| --- | --- | --- | --- | --- | --- | --- | --- |
|  | Zn | Cu | Fe |  | Zn | Cu | Fe |
| Y252/EV | 80.08±5.20 ^a^ | 6.60±0.18 ^a^ | 116.94±16.52 ^a^ |  | 76.88±1.47 ^a^ | 6.53±0.13 ^a^ | 115.15±17.79 ^a^ |
| *Δyap1*/EV | 81.37±2.74 ^a^ | 6.34±0.13 ^a^ | 114.82±29.42 ^a^ |  | 76.15±4.41 ^a^ | 6.22±0.30 ^a^ | 79.76±13.03 ^a^ |
| *Δyap1*/*NRAMP1* | 85.88±2.50 ^a^ | 6.73±0.15 ^a^ | 100.19±17.03 ^a^ |  | 83.03±4.76 ^a^ | 6.63±0.12 ^a^ | 87.17±11.05 ^a^ |

Metal contents (μg/g) were measured in the yeasts; Different letters indicate significant difference at *P* < 0.05 according to Tukey’s multiple range tests.

**Table S3 Effect of Cd treatment on metal accumulation in** ***A. thaliana* wild type and overexpression lines**

|  |  |  | Shoots |  |  |  | Roots |  |
| --- | --- | --- | --- | --- | --- | --- | --- | --- |
| Treatment | Lines | Zn | Cu | Fe |  | Zn | Cu | Fe |
| Cd0 | Col-0 | 129.3±5.93a | 6.04±0.19b | 291.0±12.77c |  | 398.3±18.82a | 21.3±2.75a | 2786±106.0bc |
|  | OE-1 | 115.0±8.15a | 7.20±0.60b | 482.0±24.19b |  | 359.3±28.94a | 18.5±2.98a | 3344±225.2b |
|  | OE-3 | 82.77±3.09b | 9.50±0.51a | 700.3±19.46a |  | 117.0±5.03c | 23.4±2.60a | 5933±23.07a |
|  | OE-4 | 81.40±2.85b | 2.83±0.27c | 340.0±15.18c |  | 214.3±10.74b | 8.36±0.76b | 2384±194.5c |
|  |  |  |  |  |  |  |  |  |
| Cd30 | Col-0 | 63.93±3.04c | 4.24±0.20a | 240.0±15.89a |  | 332.0±12.49a | 6.46±0.38a | 4995±92.5b |
|  | OE-1 | 65.77±3.70c | 2.34±0.16b | 183.7±13.04b |  | 172.3±5.24c | 4.01±0.09b | 6069±178.6a |
|  | OE-3 | 89.03±1.17b | 4.10±0.26a | 201.7±12.33ab |  | 236.7±10.41b | 3.54±0.24bc | 3790±183.1c |
|  | OE-4 | 128.7±9.70a | 4.88±0.50a | 186.7±6.36b |  | 56.97±1.73d | 2.43±0.25c | 1377±222.8d |
|  |  |  |  |  |  |  |  |  |
| Cd50 | Col-0 | 67.60±3.17b | 3.51±0.11b | 305.3±31.17b |  | 156.0±6.35b | 5.51±0.27b | 2263±194.1a |
|  | OE-1 | 53.73±2.40c | 3.45±0.19b | 525.3±16.19a |  | 82.27±2.80c | 8.52±0.65a | 2808±131.4a |
|  | OE-3 | 87.63±2.38a | 4.51±0.58b | 509.0±32.51a |  | 467.7±17.9a | 4.30±0.35c | 2652±136.5a |
|  | OE-4 | 89.63±3.060a | 8.97±0.45a | 533.7±6.89a |  | 22.3±10.49d | 5.16±0.41bc | 1000±37.54b |

Metal contents (mg/g, DW) were measured in the *A. thaliana* wild type and overexpression lines; Different letters indicate significant difference at *P* < 0.05 according to Tukey’s multiple range tests.


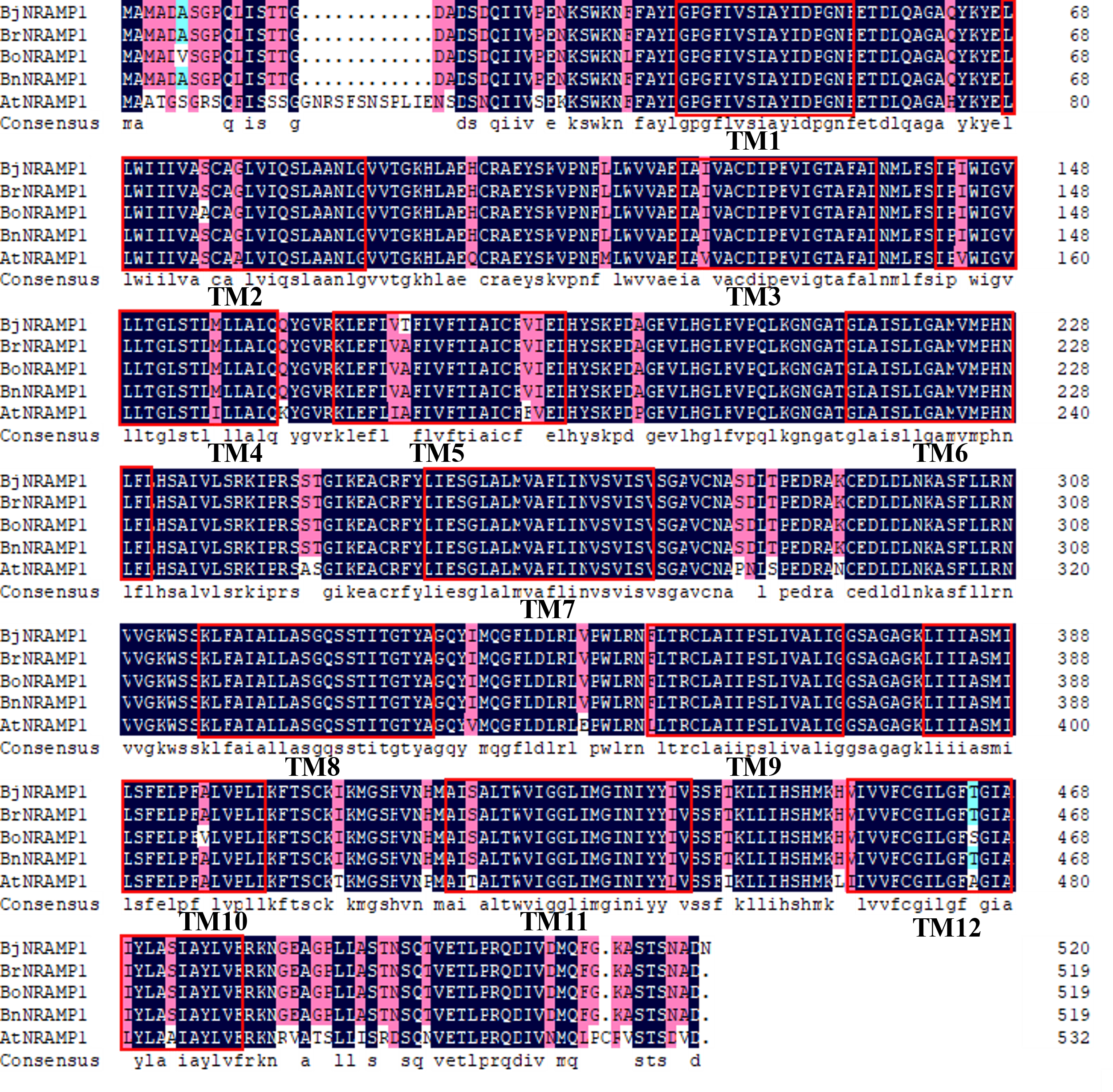


**Fig. S1. Protein sequence alignment of NRAMP1 and its homologs from several species.**

Protein sequence alignment was performed among NRAMP1 and its homologs from *Brassica rapa* (XP_009106714.1), *Brassica oleracea* ( XP_013588655.1), *Brassica napus* (XP_013726991.2) and *Arabidopsis thaliana* (NP_178198.1) using DNAMAN. The sequences marked using a red box represent the transmembrane domains which are based on the transmembrane analysis of BjNRAMP1 and predicted by TMHMM(http://www.cbs.dtu.dk/services/TMHMM/) software.


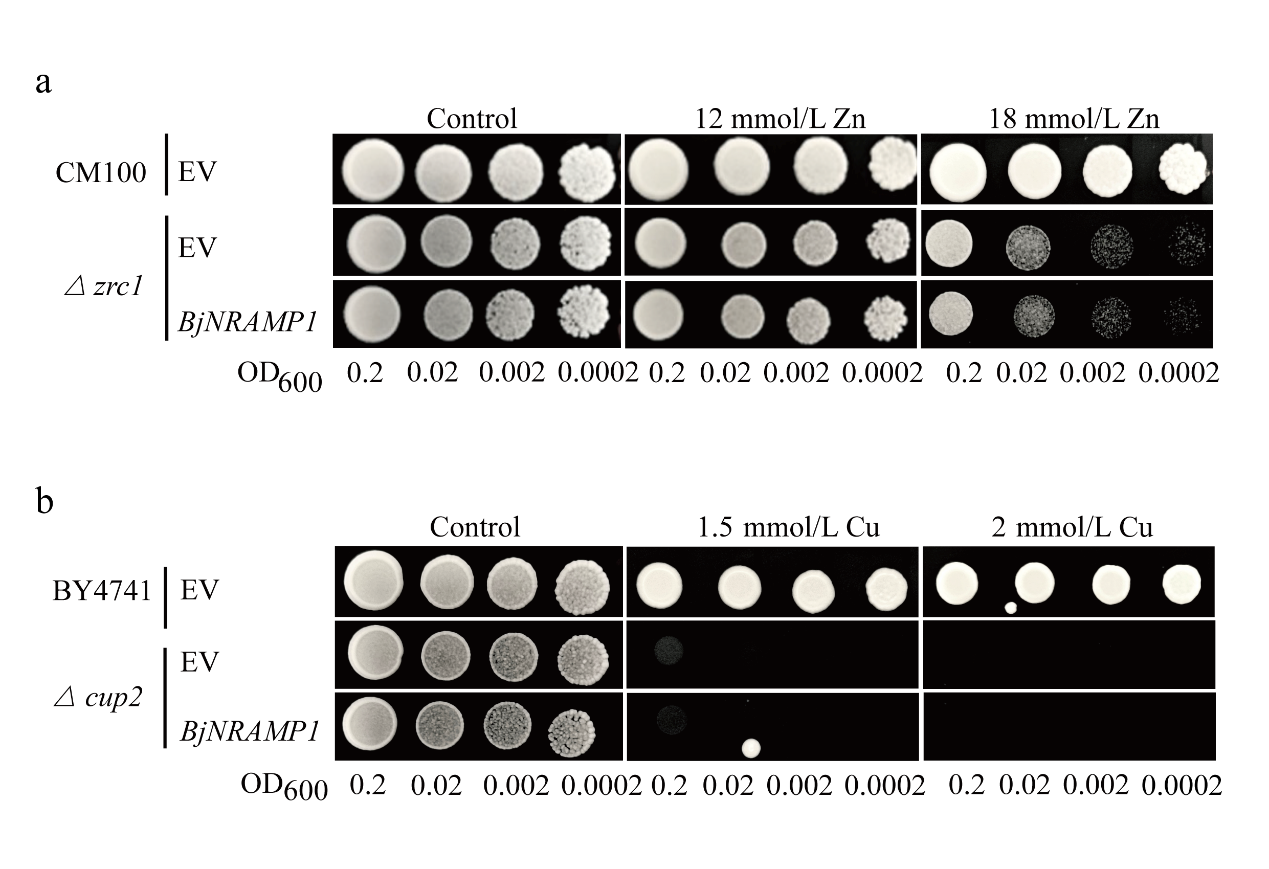


**Fig.S2. *BjNRAMP1* transport abilities in yeast.**

(a) Growth of yeast mutant strain *Δzrc1* expressing *BjNRAMP1* or empty vector (pYES2) and yeast wild strain CM100 expressing empty vector (pYES2) in a medium containing different concentrations of Zn. (b) Growth of yeast mutant strain *Δcup2* expressing *BjNRAMP1* or empty vector (pYES2) and yeast wild strain BY4741 expressing empty vector (pYES2) in a medium containing different concentrations of Cu.
